# Pregabalin modulation of spinal and brainstem visceral nociceptive processing

**DOI:** 10.1016/j.pain.2011.06.020

**Published:** 2011-10

**Authors:** Shafaq Sikandar, Anthony H. Dickenson

**Affiliations:** Department of Neuroscience, Physiology, and Pharmacology, University College London, London WC1E 6BT, UK

**Keywords:** Rostral ventromedial medulla (RVM), Visceromotor response (VMR), Colorectal distension (CRD), State-dependent actions

## Abstract

Brainstem and spinal mechanisms mediating visceral nociception are investigated here using electrophysiology and immunohistochemistry techniques in a model of acute visceral pain. Colorectal distension (CRD) produced graded visceromotor responses (VMR) in normal rats, and these were facilitated by intracolonic mustard oil (MO) that generated acute visceral hyperalgesia. The neuropathic pain drug pregabalin (PGB) is thought to have state-dependent effects in attenuating neuropathic, but not acute somatic pain, likely by impairing calcium-channel trafficking. We found that systemic PGB produced antinociceptive effects on CRD-evoked VMRs in naïve rats lacking pathophysiology and in MO-pretreated rats. Systemic PGB also significantly reduced Fos labelling in lumbosacral spinal cords of rats given noxious repetitive CRD; however, PGB did not alter this measure of neural activity in the brainstem. Differential brainstem processing of noxious somatic and visceral stimuli may underlie the unique lack of state-dependent actions of PGB in this visceral pain model. Single-unit recordings in the rostral ventromedial medulla (RVM) verify that brainstem processing of somatic and visceral stimuli differs. The effects of CRD on RVM cells classed as ON, OFF, or NEUTRAL were independent of their somatic responses, with surprising changes in RVM cell activity to innocuous visceral stimulation. PGB also markedly reduced the visceral responses of RVM ON-cells to noxious CRD. These results illustrate clear differences in the central processing of visceral and somatic stimuli, yet a common role for descending modulation by brainstem activity in mediating evoked pain measures.

## Summary

Electrophysiology and immunohistochemistry were used to investigate rat spinal and brainstem differences in somatic and visceral nociceptive processing and pregabalin efficacy in acute visceral pain.

## 1 Introduction

The relative paucity in knowledge of visceral nociceptive signalling compared to somatic pain syndromes leads to major challenges in the treatment of visceral pain disorders. A better understanding of the different processes involved in visceral nociception and an assessment of analgesic mechanisms could lead to more effective visceral pain treatments.

Numerous reports substantiate the clinical efficacy of pregabalin (PGB) in providing pain relief alone or as adjunctive therapy in neuropathic pain patients [1,2,34,39]. However, the analgesic potential of gabapentinoids in other pain states, including fibromyalgia, postoperative pain, and osteoarthritis, has been supported by animal and clinical studies [13,34,40]. A clinical trial with irritable bowel syndrome patients suffering from rectal hypersensitivity showed that PGB increased distension sensory thresholds to normal levels [26].

Binding to the ubiquitous α_2_δ subunit of voltage-gated calcium channels is both necessary and sufficient for the analgesic effects of gabapentanoids, and this may impair channel trafficking [3,14,19,25,28]. In addition, pathological states and resultant interactions with a spino-bulbo-spinal loop comprising projection neurones in the superficial dorsal horn and brainstem descending serotonergic facilitations mediated by 5-HT_3_ receptors are permissive for gabapentanoid analgesia [5,46]. Together, these state-dependent changes allow PGB to attenuate neuropathic, but not acute, somatic pain.

Disordered descending modulation from the brainstem may underlie abnormal pain perception in functional somatic and visceral pain disorders [37,51,52]. Functional imaging shows activation of the brainstem rostral ventromedial medulla (RVM) during both visceral and somatic pain [16]. The RVM plays a key role in central pain modulation and is an important relay for integration of descending influences onto the spinal cord. RVM neurones are classified based on their firing patterns to noxious somatic stimuli [20]. ON-cells increase firing immediately before a nocifensive response and are presumably pronociceptive; OFF-cells are thought to mediate inhibition and transiently pause firing prior to a nocifensive response; NEUTRAL-cells show no consistent change in firing to a noxious somatic stimulus, and their role in nociceptive processing yet remains unclear.

In models of acute visceral pain, electrical stimulation of the RVM produces intensity-dependent facilitation or inhibition of spinal neuronal responses to colorectal distension (CRD) [54,55]. The maintenance of pancreatitis-induced abdominal hypersensitivity is prevented by selective ablation of pronociceptive RVM cells, likely ON-cells [53]. The RVM can therefore promote excitability that produces visceral pain through mechanisms that may correspond to the descending RVM facilitations that contribute to the analgesic actions of PGB [5,46].

We use electrophysiological recordings to compare responses of RVM neurones to somatic and visceral CRD stimuli to determine whether brainstem processing, and thereby generation of descending influences on spinal excitability, differ between somatic and visceral signalling. We also investigate whether PGB has analgesic efficacy in the acute visceral pain model of CRD with and without a transient visceral hyperalgesia, and we further use RVM recordings and immunohistochemistry to determine whether and how systemic administration of PGB alters excitatory neural activity centrally in the spinal cord and in the brainstem following CRD. These studies will assess whether state-dependent actions of PGB in neuropathy also apply to acute visceral pain.

## 2 Methods

All studies used male Sprague–Dawley rats (250–300 g) supplied by Biological Services Unit (BSU, University College London, UK). All procedures were approved by the Home Office (UK) and were in agreement with the International Association for the Study of Pain guidelines [56].

### 2.1 Colorectal distension

Rats were initially anaesthetized in an induction box with 4% isoflurane in a mixture of N_2_O (66% v/v) and O_2_ (33%). A tracheotomy was performed once the rats were unconscious and areflexic. The trachea was exposed and a tracheal cannula was inserted and tied with 3-0 silk thread. Anaesthesia was maintained through the tracheal cannula throughout the recording period. Rats were secured to a stereotaxic frame with a heating blanket to maintain a core temperature of 37 °C.

The balloon for CRD was made using 7 cm of a latex balloon tied with silk thread to a cannula perforated throughout a 5-cm tip, and inserted 1 cm intra-anally. Isoflurane was maintained at 1% v/v for the remainder of the recording period, a level of anaesthesia sufficient to prevent spontaneous movements while maintaining reflex motor controls. CRDs were produced by inflating the balloon through a pressure amplifier to measure the degree of inflation as mm Hg.

### 2.2 Electromyography recordings

Isoflurane was maintained at 1.5% v/v while an incision was made in the right lower quadrant of the abdomen to expose underlying muscle. An enamel-coated copper electrode was sown into the right external oblique muscle and the incision was sutured with skin clips.

The recording electrode was inserted into a head stage as part of a Neurolog system with a 1401 interface and Spike4 software (Cambridge Electronic Design, Cambridge, UK). Captured signals from muscle activity were amplified, filtered, and displayed on an oscilloscope, and signals were further integrated to produce electromyography (EMG) values.

CRDs were given in increasing steps of 10 mm Hg from 10 mm Hg to 80 mm Hg, lasting for 30 seconds, with 3-minute intervals between each distension and 15-minute intervals between series. The mean integrated EMG values evoked during CRD (visceromotor responses [VMRs]) were used for further analysis.

Once the average control EMG values were obtained for distensions from 10 mm Hg to 80 mm Hg, PGB was administered subcutaneously (30 mg/kg) and series of distensions were performed at 20 and 60 minutes later and evoked VMR recorded.

Reproducibility of the CRD model and evoked VMRs was validated in preliminary studies by confirming that the evoked VMR EMG values did not differ more than 15% from the average EMG control value at 20 and 60 minutes after the EMG control value, corresponding to PGB administration time points.

### 2.3 Visceral hyperalgesia

Rats remained anaesthetized with isoflurane (1.5%) in a mixture of N_2_O (66% v/v) and O_2_ (33% v/v). A cannula was used to administer 1 mL of mustard oil (MO; 0.25% in mineral oil) 1–6 cm intra-anally.

The isoflurane level was reduced to that used in control EMG recordings, and 30 minutes were allowed for visceral hyperalgesia to develop before resuming EMG recordings. PGB was administered subcutaneously (30 mg/kg) 10 minutes after MO. Series of distensions were performed and evoked VMR was recorded 20 and 60 minutes after PGB administration (corresponding to 30 and 70 minutes after MO application).

### 2.4 c-Fos expression

All rats were anaesthetized and a tracheotomy was performed to maintain anaesthesia with isoflurane in a mixture of N_2_O (66% v/v) and O_2_ (33% v/v) for the duration of the experiment, and a CRD balloon was made and inserted and isoflurane was lowered to 1.0% v/v (see Section 2.1 details).

For the noxious CRD protocol, the balloon was distended to 70 mm Hg for 2 hours (30 seconds on, 90 seconds off). This paradigm has been shown to result in a significant expression of *c-Fos* in the lumbosacral cord [50]. For the noxious + PGB protocol, rats were administered PGB (30 mg/kg subcutaneously) 5 minutes prior to the 2-hour Noxious distension protocol. Sham rats had CRD balloons inserted but no distensions given.

At 2 hours following the end of the CRD protocol for *c-Fos* induction, rats were deeply anesthetized with 1 mL (200 mg intraperitoneally) pentobarbitone sodium (Merial Animal Health Ltd, Harlow, Essex, UK). The thoracic cage was cut open to expose the heart, and animals were transcardially perfused with 300 mL saline (0.9% w/v) in solution with heparin (5000 IU/L saline) (LEO Laboratories, Buckinghamshire, UK). This was followed by 300 mL of 4% paraformaldehyde (VWR, Lutterworth, Leicestershire, UK) in 0.6 M phosphate-buffered solution (PBS).

A laminectomy was performed to expose and remove spinal cord segments L5-S2, and a craniotomy was performed to expose and remove the hindbrain. Tissue was postfixed for 2 hours before being transferred to a cryoprotectant solution of 30% w/v sucrose in 0.1 M PBS and 0.01% w/v sodium azide (Sigma Aldrich, Dorset, UK) for a minimum of 3 days.

All tissue was cut on a freezing microtome in 40-μm sections, collected serially and stored in a cryoprotectant solution of 5% w/v sucrose in 0.1 M PBS and 0.01% w/v sodium azide. Tissue sections were transferred to Fluorescence-activated cell sorting (FACS) tubes containing blocking solution made of 3% normal goat serum in 0.1 M PBS with 10% v/v triton x-100 (Sigma Aldrich) and H_2_O_2_ (VWR) and incubated for 1 hour. The blocking solution was replaced with the rabbit polyclonal *c-Fos* primary antibody (1:20,000 brain sections, 1:60,000 spinal cord sections) (Calbiochem, Nottingham, UK) and the tissue was incubated for 24 hours at room temperature.

Tissue sections were then washed to remove excess primary antibody and incubated in biotinylated secondary antibody (Goat Anti-Rabbit, 1:500 in Tween/Tris-buffered saline (TTBS)) for 2 hours. Following another round of washes, tissue sections were incubated in ABC (1:1000 TTBS Vectastain A, 1:1000 Vectastain B) for 1 hour. Tissue sections were washed again to remove excess AB complex, and conventional 3,3′-diaminobenzidine (DAB) staining (DAB substrate kit, Vector Laboratories, Burlingame, CA, USA) in the presence of hydrogen peroxide was used to reveal Fos immunoreactivity. Accordingly, tissue sections were incubated in DAB for 10–15 minutes before being transferred to distilled water for 5 minutes to stop the peroxidase reaction. Tissue sections were transferred to 0.1 M PBS before being mounted onto gelatinized slides. Slides were allowed to dry overnight, and were dehydrated the following day, coverslipped with DPX mounting agent (VWR), and sealed with nail varnish.

### 2.5 RVM recordings

Following the tracheotomy (see CRD details), the skull was exposed to mark positions of lambda and bregma. The incisor bar was adjusted (3.9 ± 0.5 mm) below horizontal zero until heights of bregma and lambda were equal to achieve the flat skull position. An area correlating to the RVM was marked and drilled 2 mm in diameter and the dura removed.

A CRD balloon was made and inserted. Isoflurane was slowly lowered to 1.2% v/v with O_2_ alone over a 1-hour period. Once the rats were stabilized at an anaesthetic level sufficient to maintain reflex withdrawal from pinching the paw and tail, a recording electrode (parylene-insulated tungsten microelectrode, 12-μm diameter, 2 MΩ; A-M Systems Inc, Carlsborg, WA, USA) was inserted into the RVM (0.0–0.9 mm mediolateral, 10.5–11.5 mm caudal from bregma, 9.0–11.0 mm dorsal from dura matter).

The recording electrode was inserted into a head stage as part of a Neurolog system with a 1401 interface and Spike4 software (Cambridge Electronic Design). Neuronal activity was amplified, filtered, and displayed on an oscilloscope and made audible through speakers. Single neurones were isolated based on good signal-to-noise ratios and common action potential shapes. RVM cells were identified as ON, OFF, or NEUTRAL according to their change in firing following a noxious thermal stimulus (55 °C immersion of 8 cm of the tail), in terms similar to those described by Fields et al. [20]. The relative change in firing induced by noxious heat was measured as spikes following the reflex tail flick subtracted by baseline firing (5 seconds of activity after the tail flick minus 5 seconds of activity prior to the tail flick). The classification of ON-, OFF-, and NEUTRAL-cells was based on a >15% change in baseline firing.

Once an RVM cell had been characterised according to somatic responses with tail heat, its responses to CRDs were determined. Again, changes >15% of the baseline firing determined whether cells were classified as ON, OFF, or NEUTRAL to visceral stimulation (20 seconds of activity during CRD subtracted by 20 seconds of activity prior to CRD). CRDs were given as innocuous (20 mm Hg) or noxious (70 mm Hg) visceral stimuli lasting 20 seconds, with a 3-minute interval between each distension. A pressure amplifier converted signals from the distending balloon to mm Hg. The relative numbers of spikes evoked during the 20-second CRD period subtracted by a 20-second baseline were used for further analysis.

### 2.6 Statistical analysis

EMG values were integrated with subtracted baselines and postdrug data were normalized for each animal to the mean control EMG values. Normalized EMG data were evaluated with both the Kolmogorov–Smirnov (with Dallal–Wilkinson–Lilliefor *P* value) and the D’Agostino and Pearson omnibus normality tests to confirm that data were normally distributed and therefore eligible for analysis of variance (ANOVA) with Bonferroni post-tests. A 2-way ANOVA with repeated measures and Bonferroni post-tests was used to determine statistical significances throughout the range of CRD pressures between the 3 recording time points for the PGB time course in normal rats and MO-treated rats. For area-under-curve analysis, a 1-way ANOVA with Bonferroni post-tests was used to determine statistical differences between groups. Similarly, a 2-way ANOVA with repeated measures and Bonferroni post-tests was used to determine the statistical significances between noxious or innocuous CRD-evoked RVM neuronal activity throughout the PGB time course. A 1-way ANOVA with Bonferroni post-tests was used to determine statistical differences among evoked activities during the 4 recording points in the PGB time course.

For Fos immunohistochemistry, analysis of labelled neurones in both brain and spinal cord sections involved 4 animals in each group (Sham distension, Noxious distension, Noxious distension with PGB, and Sham distension with PGB) and 5 tissue sections per animal. A 1-way ANOVA with Bonferroni post-tests was used to determine the significant difference between numbers of Fos-labelled neurones quantified in the 3 groups using both RVM and L6-S1 sections.

## 3 Results

### 3.1 Validation of CRD model and pregabalin modulation of VMRs

**Fig. 1:**
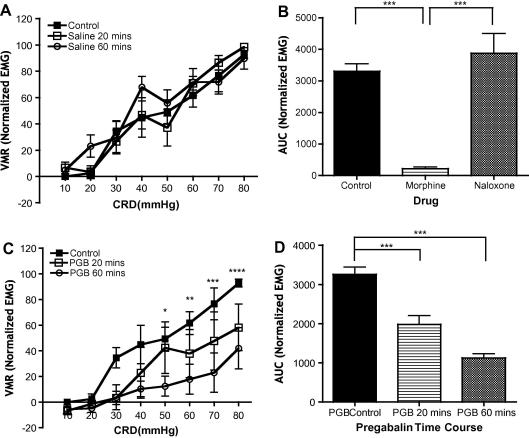
Pregabalin (PGB) reduces visceromotor response (VMR) throughout the noxious range of colorectal distension (CRD) pressures in naïve rats. (A) Line graphs representing evoked VMRs by a range of innocuous to noxious CRD pressures to illustrate the stability and reproducibility of VMRs. There is no significant change in VMR between baseline control and following control saline administration at 20 and 60 minutes (n = 5). (B) Area-under-curve bar graphs of overall evoked VMRs in naïve rats (n = 9) illustrate the dramatic reduction in VMR by the full range of CRD pressures by morphine (2.5 mg/kg subcutaneously [s.c.]), and the reversal of this morphine-induced inhibition by naloxone (2 mg/kg s.c., ^∗∗∗^*P *< 0.001). (C) Line graphs representing evoked VMRs by a range of innocuous-to-noxious CRD pressures for the 3 recording points in the PGB time course in naïve rats (n = 6; 30 mg/kg s.c.). Sixty minutes after PGB administration, the evoked VMR is reduced dramatically over the 30-mm-Hg to 80-mm-Hg CRD pressures, corresponding to the noxious range of colonic distension (^∗^*P *< 0.05, ^∗∗^*P *< 0.01, ^∗∗∗^*P *< 0.001 control vs. 60 minutes). (D) Area-under-curve comparisons of the evoked VMR illustrate an overall significant reduction throughout both the 20- and 60-minute recordings after PGB administration (^∗∗∗^*P *< 0.001).

A reproducible model of visceral pain is illustrated by the graded evoked VMRs following increasing CRD pressures. Stability of VMRs is demonstrated using saline injections with VMRs measured 20 and 60 minutes after administration (Fig. 1A).There is no significant change in VMR during this recording period (time variable F[2,80] = 1.539, *P *> 0.05; pressure variable F[7,80] = 29.79, *P *< 0.0001). Moreover, we also show that the VMR can be pharmacologically manipulated (Fig. 1B). Morphine dramatically reduces VMR evoked by the full range of CRD pressures, and this morphine-induced inhibition is reversed by administration of naloxone (F[2,11] = 25.86, *P *< 0.0001).

Control-evoked VMRs show clear, graded responses with magnitudes correlating to increasing CRD pressures from 10 mm Hg to noxious CRD of 80 mm Hg (Fig. 1C). Systemic administration of PGB in naïve rats dramatically reduces the evoked VMR throughout the noxious CRD pressure range of 30 mm Hg to 80 mm Hg CRD stimuli at 20 minutes and further at 60 minutes post-PGB (Fig. 1D); (F[2,15] = 67.66, *P *< 0.0001).

### 3.2 PGB modulation of visceral hyperalgesia

**Fig. 2:**
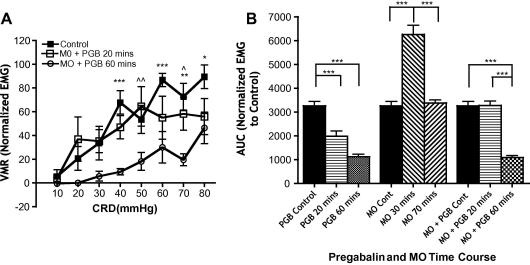
Pregabalin prevents development of visceral hyperalgesia induced by intracolonic mustard oil (MO). (A) Line graphs representing evoked visceromotor responses (VMRs) by a range of innocuous to noxious colorectal distension (CRD) pressures for the 3 recording points in the pregabalin (PGB) time course (30 mg/kg subcutaneously) in rats pretreated with intracolonic MO to induce visceral hyperalgesia (n = 7; ^∗^*P *< 0.05, ^∗∗^*P *< 0.01, ^∗∗∗^*P *< 0.001 control vs. 60 minutes; ^^^*P *< 0.05, ^^^^*P *< 0.01 20 vs. 60 minutes). (B) Collective area-under-curve data of groups with PGB alone (n = 6), with intracolonic MO alone (n = 8), and with PGB and intracolonic MO pretreatment (n = 7). Rats with intracolonic MO show a transient potentiation of evoked VMR that is lost in rats also given systemic PGB (^∗^*P *< 0.05, ^∗∗^*P *< 0.01, ^∗∗∗^*P *< 0.001).

PGB also prevents the development of potentiated VMRs in rats pretreated with intracolonic MO (Fig. 2A) (time variable F[2,64] = 33.88, *P *< 0.0001; pressure variable F[7,64] = 7.358, *P *< 0.0001). Visceral hyperalgesia is illustrated by a time-dependent potentiation of evoked VMR peaking at 30 minutes post-MO and declining to control VMR values at 70 minutes post-MO (Fig. 2B) (F[8,9] = 55.88, *P *< 0.0001). There is no significant difference between control VMRs and VMRs evoked at the 20-minute recording point in the MO + PGB group at any CRD pressure, illustrating a complete inhibition of the potentiation response by PGB. Moreover, PGB dramatically reduces the evoked VMR at the 60-minute MO + PGB time point, illustrating long-lasting inhibitory actions of PGB as in naïve animals. Thus, PGB has inhibitory actions on noxious evoked VMRs in normal animals and in animals with visceral hypersensitivity.

### 3.3 c-Fos expression following repetitive noxious CRD

**Fig. 3:**
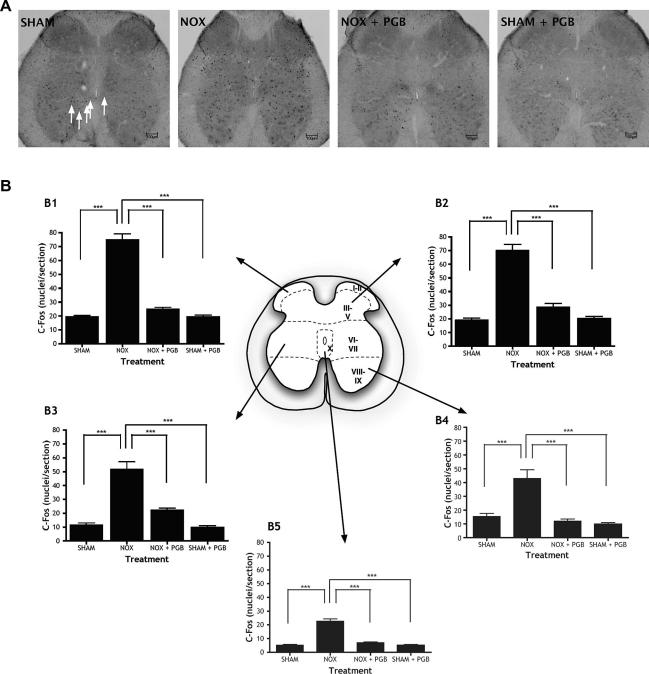
*c-Fos* expression is higher following noxious colorectal distension (CRD) in L6-S1 and is significantly reduced by systemic pregabalin (PGB). (A) Representative photomicrographs of L6-S1 spinal cord sections in the 4 treatment groups: sham CRD (SHAM), noxious CRD (NOX) and noxious CRD with PGB (NOX + PGB; 30 mg/kg s.c.) and sham CRD with PGB (SHAM + PGB; 30mg/kg s.c.). Arrows indicate Fos-labelled neurones. (B) Numbers of *c-Fos*-labelled neurones in different regions of L6-S1 spinal cord tissue of sham CRD (SHAM), noxious CRD (NOX), noxious CRD with PGB (NOX + PGB) and sham CRD with PGB (SHAM + PGB) rats are illustrated for different regions of the spinal cord (superficial dorsal horn: laminae I–II [B1], deep dorsal horn: laminae III–V [B2], intermediate region: laminae VI–VII [B3], ventral horn: laminae VIII–IX [B4], central canal: lamina X [B5]). Noxious CRD markedly increases *c-Fos* labelling in all quantified regions of the spinal cord compared to innocuous CRD, and this is significantly reduced by PGB in throughout the dorsal and ventral regions (^∗^*P *< 0.05, ^∗∗∗^*P *< 0.001). Scale bars = 100 μm.

**Fig. 4:**
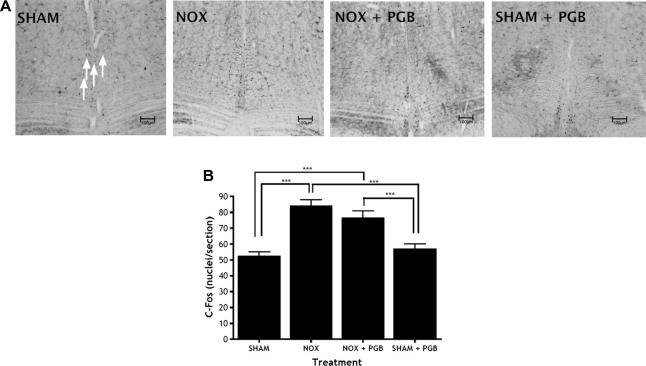
Levels of *c-Fos* expression are higher following noxious colorectal distension (CRD) in the rostral ventromedial medulla (RVM) but are unchanged after systemic pregabalin (PGB). (A) Representative photomicrographs of RVM sections in the 4 treatment groups: sham CRD (SHAM), noxious CRD (NOX), and noxious CRD with PGB (NOX + PGB; 30 mg/kg and sham CRD with PGB (SHAM + PGB; 30mg/kg s.c.). Arrows indicate *c-Fos*-labelled neurones. (B) Numbers of *c-Fos*-labelled neurones in RVM tissue of the sham CRD (SHAM), noxious CRD (NOX), noxious CRD with PGB (NOX + PGB) and sham CRD with PGB (SHAM + PGB) rats. Noxious CRD dramatically increases *c-Fos* cell counts, yet this level of *c-Fos* expression is unchanged with PGB administration (^∗∗∗^*P *< 0.001). Scale bars = 100 μm.

Noxious CRD markedly increases the number of *c-Fos*-labelled neurones compared to sham CRD both in L6-S1 spinal cord sections (Fig. 3) and in brainstem RVM sections (Fig. 4). In the spinal cord, the increase in Fos immunoreactivity is generalized throughout the dorsal and ventral horns (lamina I–II F[3,16] = 114.1, *P *< 0.0001; lamina III–V F[3,16] = 74.75, *P *< 0.0001; lamina VI–VII F[3,16] = 37.86, *P *< 0.0001; lamina X F[3,16] = 79.36, *P *< 0.0001). This would be expected since sensory neurones in the dorsal horn would be activated by visceral afferent activity, in turn activating ventral horn neurones that are responsible for the observed muscular contractions. PGB administration significantly reduces the overall noxious CRD-induced increase in Fos counts in the spinal cord effectively to control values. In contrast, PGB does not alter Fos immunoreactivity in the RVM (F[3,16] = 15.55, *P *< 0.0001). In both RVM tissue and spinal cord tissue, there is no significant difference between the SHAM group and the SHAM + PGB group, so PGB is only reducing noxious-evoked activity.

### 3.4 Physiological responses of RVM neurones to somatic and visceral stimuli and PGB effects on RVM neural activity

**Fig. 5:**
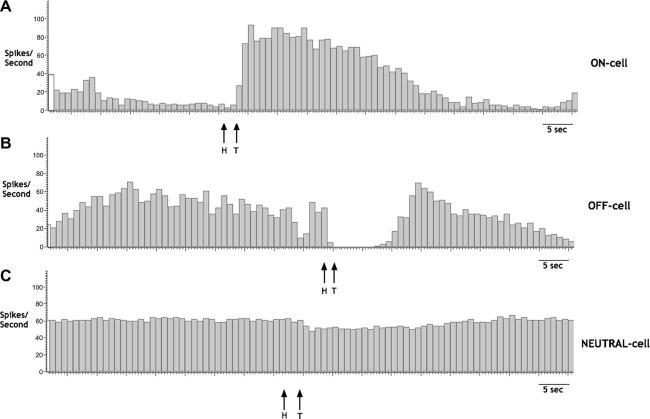
Rostral ventromedial medulla (RVM) ON-, OFF-, and NEUTRAL-cells display characteristic changes in reflex-related firing following a noxious somatic stimulus. (A) Example trace of an RVM ON-cell that increases reflex-related firing following noxious tail heat (H = onset of heat stimulus, T = tail flick). (B) Example of a ratemeter trace of an RVM OFF-cell that pauses reflex-related firing (spikes) following noxious tail heat. (C) Example trace of a NEUTRAL-cell that displays no consistent change in reflex-related activity following noxious tail heat.

**Fig. 6:**
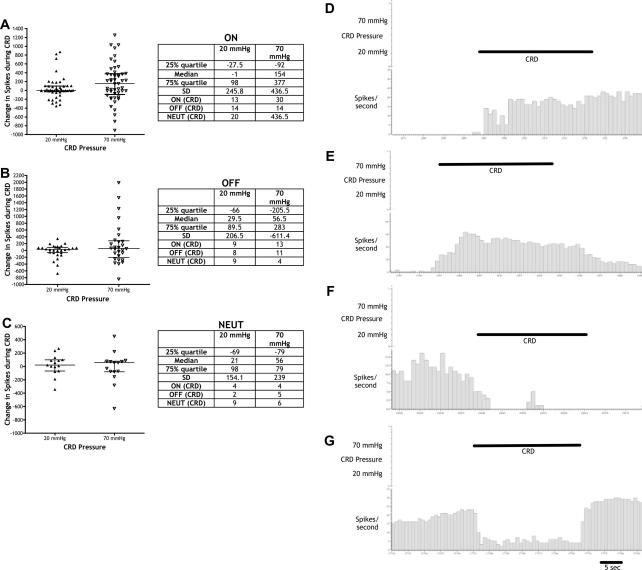
The responses of rostral ventromedial medulla (RVM) ON-, OFF-, and NEUTRAL-cells to noxious somatic stimuli do not predict changes in reflex-related firing following innocuous and noxious visceral stimuli. (A) Changes in activity of RVM ON-cells during innocuous (20 mm Hg) and noxious (70 mm Hg) colorectal distension (CRD), measured as number of spikes differing from baseline (n = 44). (B) Changes in activity of RVM OFF-cells during innocuous (20 mm Hg) and noxious (70 mm Hg) CRD (n = 17). (C) Changes in activity of RVM NEUTRAL-cells during innocuous (20 mm Hg) and noxious (70 mm Hg) CRD (n = 14). Upper and lower quartiles, median number of spikes, and standard deviation are noted with the graphs. Numbers of ON, OFF, and NEUTRAL-cells behaving in an ON, OFF, or NEUTRAL-like fashion to CRD stimulation are also noted. (D) An example ratemeter trace of an OFF-cell that increases firing to innocuous CRD and (E) to noxious CRD. (F) An example ratemeter trace of an ON-cell that increases firing to innocuous CRD and (G) to noxious CRD.

Electrophysiological recordings in the brainstem illustrate that RVM ON-, OFF-, and NEUTRAL-cells show characteristic changes in reflex-related firing following the onset of the noxious somatic heat stimulus to tail (Fig. 5). Accordingly, ON-cells increase reflex-related firing following noxious tail heat; OFF-cells pause reflex-related firing following noxious tail heat; NEUTRAL-cells show no consistent change in reflex-related activity following noxious tail heat. In contrast, the recordings of neuronal activity during visceral CRD stimuli do not display changes consistent with this somatic-based classification, as RVM ON-, OFF-, and even NEUTRAL-cells display changes in activity to innocuous (as well as noxious) visceral stimulation, and also variably increase, decrease, or show no change in firing during CRD (Fig. 6). The RVM neural processing of somatic and visceral stimuli must thus differ. The changes in cell firing are greater during noxious CRD stimulation compared to innocuous CRD. Accordingly, more ON-, OFF-, and NEUTRAL-cells responded to noxious CRD compared to innocuous CRD. Ninety percent of ON-cells, 89% of OFF-cells, and 60% of NEUTRAL-cells display >15% changes in baseline spike activity during 70-mm-Hg CRD, compared to 55%, 71%, and 40%, respectively, during 20-mm-Hg CRD. The greater variability of responses during noxious CRD stimulation reflects the increased magnitudes of firing of ON-, OFF-, and NEUTRAL-cells compared to innocuous CRD.

**Fig. 7:**
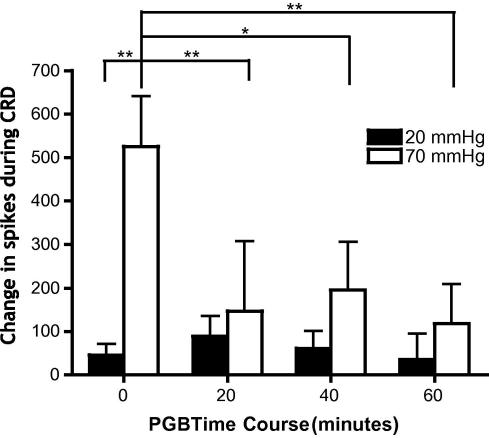
Pregabalin (PGB) inhibits noxious colorectal distension (CRD)-evoked activity of rostral ventromedial medulla (RVM) ON-cells that are also excited by noxious CRD, but does not affect the innocuous CRD-evoked activity. Recordings made from RVM ON-cells that increase firing to both noxious somatic and innocuous and noxious CRD visceral stimulation (n = 11). Bar graphs illustrate the change in activity during innocuous (20 mm Hg) and noxious (70 mm Hg) CRD of RVM ON-cells before and after systemic PGB administration (30 mg/kg subcutaneously) (^∗^*P *< 0.05, ^∗∗^*P *< 0.01).

Thus, control responses of RVM ON-cells during noxious CRD prior to PGB administration at time 0 are significantly greater than responses to innocuous CRD (Fig. 7) (F[3,60] = 4.402, *P *< 0.01). Following PGB administration, the firing of RVM ON-cells during noxious CRD is dramatically inhibited throughout the 20-, 40-, and 60-minute recording periods (F[3,7] = 7.493, *P *< 0.0001). This is similar to the 60-minute time frame of PGB reductions of overall evoked VMR in naïve rats. However, PGB does not have any significant inhibitory effect on the responses of ON-cells during innocuous CRD (F[3,7] = 1.367, *P *> 0.05).

**Fig. 8:**
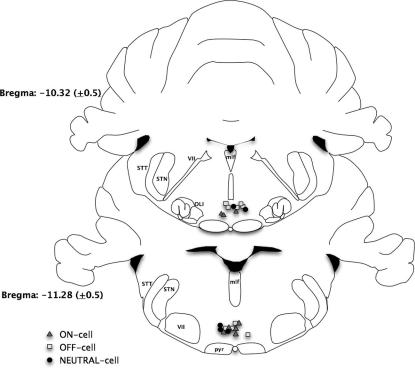
Rostral ventromedial medulla (RVM) recording sites. A diagrammatic representation of coronal sections corresponding to RVM nuclei with recording sites of ON-, OFF-, and NEUTRAL-cells indicated. Coronal depth is evaluated by the dorsoventral distance from the horizontal plane passing through bregma and lambda on the surface of the skull. Every fifth ON-cell and every third OFF- and NEUTRAL cell have been indicated for clarity due to large *n* numbers in each group. All recorded cells used for analysis lie within RVM anatomical boundaries.

Only the effects of PGB on ON-cell activity were fully analyzed. Despite strenuous efforts, we were unable to record, for sufficiently long periods, OFF-cell responses to visceral stimuli. However, PGB did reduce the magnitude of the pause in activity of 2 medullary cells that had OFF-like responses to CRD. Cellular recording sites were stereotaxically defined (Fig. 8).

## 4 Discussion

Graded visceromotor responses to CRD measured with EMG recordings are attenuated by systemic PGB both in normal animals and in rats with MO-induced hypersensitivity at doses that are effective in neuropathic models [5]. Marked differences in nociceptive modulation of somatic structures and viscera are illustrated by this efficacy of PGB in reducing acute visceral nociception, compared to its state-dependent actions in neuropathy, where PGB has little effect in naïve animals but attenuates pain measures in animals with somatic pathology. We also show a differential brainstem processing of somatic and visceral sensory information, where the definition of RVM ON-, OFF-, and NEUTRAL-cells (by somatic stimuli) does not predict their responses to visceral innocuous and noxious CRD stimuli. PGB attenuates excitatory responses of RVM neurones to noxious visceral stimuli.

Systemic PGB significantly reduces evoked visceral pain in normal rats and in rats with a transient visceral hyperalgesia, confirming the significant reduction of overall CRD-evoked EMG by a range of distensions (10–80 mm Hg) reported previously following PGB administration [41]. This nociceptive-specific efficacy of PGB correlates with previous behavioural findings of similar systemic doses of PGB reducing evoked VMRs following noxious CRD [18,33].

PGB likely exerts analgesic effects in the CRD model by modulation of calcium channel function, although interactions with upregulated calcium channel subunits after neuropathy [30] may not extend to all pain states. Gabapentanoids exert inhibitory actions in short-term inflammatory models [18,43] and the CRD model presented here, where α_2_δ upregulation has insufficient time to occur. In contrast, gabapentanoids do not change spinal neuronal responses to nociceptive mechanical or thermal somatic stimuli in normal animals, but are efficacious in chronic pain models of neuropathy and osteoarthritis [5,40,46]. This minimal modulation of normal synaptic transmission in the dorsal horn [17,40,46], but modulation of central-hyperalgesic states [15,27,43] is supported by functional magnetic resonance imaging data showing that gabapentin has a state-specific modulation of brainstem activation only in the presence of central sensitisation [27]. Sufficient central excitability as a prerequisite for the analgesic efficacy of PGB could develop as central sensitisation following persistent or sufficient peripheral afferent barrage into the CNS [12]. Although central sensitisation is mainly described as a spinal cord phenomenon involving *N*-methyl-d-aspartate receptor activity, supraspinal structures also contribute to its development and maintenance [12,52].

As visceromotor responses to acute CRD stimuli are produced by lumbosacral-bulbo-spinal loops [35], consequent spinal and brainstem activation may produce a permissive state of central excitability for systemic PGB to exert inhibitory actions in naïve rats or in rats with acute inflammation [18,33,41].

Further evidence for differential central processing of visceral and somatic stimuli is the contrasting changes in brainstem activity to visceral and somatic stimulation. Functional magnetic resonance imaging reveals equivalent bilateral activation of the periaqueductal grey or RVM following somatic and visceral stimulation [16]. This highlights a role for the RVM in nociceptive processing of both somatic and visceral sensory transmission in humans. Importantly, our electrophysiological recordings of RVM neurones in the CRD model reveal that reflex-related activity of ON-, OFF-, and NEUTRAL-cells to noxious somatic stimuli is not predictive of changes in activity of these cells during visceral stimulation [20]. The findings that RVM neurones do not respond in the same direction to visceral and cutaneous stimulation, but show similar changes in absolute magnitude, is consistent with previous observations of RVM cell responses to CRD in rats [8,9] and to bladder stimulation in the cat [10]. We have also shown that the ambiguous role of NEUTRAL-cells in modulating somatic noxious signalling is contrasted by direct increases or decreases in reflex-related firing of these cells following visceral stimulation.

Therefore, RVM neurones can have independent, multi-directional responses to and effects on visceral and somatic nociceptive processing. This may underlie spinal mechanisms of viscerosomatic convergence as well as heterotopic antinociception demonstrated by CRD-evoked suppression of cutaneous withdrawal reflexes [7].

Surprisingly, in contrast to responding almost exclusively to noxious cutaneous stimuli in normal animals [20,29], these RVM neurones also displayed changes in activity to innocuous 20-mm-Hg CRD, a nonaversive visceral stimulus in awake rats [8,9]. The responses of RVM neurones to innocuous visceral stimulation may be related to distension pressure coding of low-threshold mechanosensitive visceral afferents that also code noxious stimuli [42]. Importantly, this elaborated heterogeneity of RVM neurones emphasizes differences in the central neuronal and, specifically, brainstem processing of somatic and visceral signals, which may further contribute to different physiological prerequisites for the analgesic efficacy of PGB in somatic and visceral pain states.

We showed that firing of a subset of RVM ON-cells, specifically neurones that also increased firing to CRD, was reduced with systemic PGB. Because these ON-cells increase firing to both somatic and visceral stimulation, they likely have a pronociceptive, facilitatory role in both somatic and visceral nociceptive processing. This supports numerous findings of pronociceptive RVM ON-cell activity [4,23,24,36].

*c-Fos* expression in the spinal cord and RVM following noxious repetitive CRD was reduced throughout the ventral and dorsal horns and central canal by PGB. These spinal cord regions are all innervated by the RVM [21], although the parallel reduction in ventral and dorsal horn Fos labelling is likely to indicate a primary PGB depression of sensory neurotransmission in the dorsal horn that secondarily drives a reduction in ventral horn neuronal activity and motor output. This corresponds with other electrophysiological studies reporting reductions in sensory-evoked responses of spinal neurones of animals with neuropathy following PGB administration [5]. Here again, PGB is effective in reducing visceral Fos labelling in normal animals.

The PGB effects on RVM ON-cell neuronal activity, yet the lack of change in RVM Fos-labelling, could be explained by Fos labelling not being able to distinguish between neural activity related to nociceptive processing and antinociception [22]. These results can be explained by different subsets of RVM neurones producing differential pro- and antinociceptive effects that cannot be distinguished by Fos. Furthermore, Fos-negative neurones could encode significant amounts of nociceptive information in the brainstem. Thus, if PGB increased OFF- and decreased ON-cell activity in equal proportions, there would be consequent antinociceptive effects on spinal processing and inhibition of evoked VMRs, yet no change in labelling would be seen. We show that systemic PGB reduces evoked neural activity in the spinal cord and excitatory RVM neuronal activity, although its direct central sites of action are unknown. Thus, RVM effects could be secondary to altered activity at spinal levels.

We characterized RVM neurones using isoflurane and oxygen, and RVM cell types corresponded in all regards to those described by others using halothane or pentobarbital and methohexital [8,11]. The addition of N_2_O during *c-Fos* induction could have affected results, but we observed a clear inhibition of both EMG and Fos labelling at the spinal level. Thus, any effect of N_2_O would have to be selective for RVM, but as stated above, we saw clear inhibitory effects of PGB on ON-cell activity at this level.

In models of neuropathy and ostearthritis, gabapentanoid modulation depends on the permissive pathological state involving specific interactions with descending serotonergic facilitations mediated by brainstem-spinal mechanisms and hypothesized interplay between presynaptic 5-HT_3_ receptors and α_2_δ subunits of voltage-gated calcium channels [5,46]. This is substantiated by the upregulation of α_2_δ_1_ subunits in ipsilateral dorsal root ganglia in neuropathy and osteoarthritis [3,40], with concomitant serotonergic 5-HT_3_ receptor-mediated facilitations that both increase spinal excitability and allow gabapentanoid actions [40,45].

In models of peripheral nerve injury, spinal and supraspinal administration of gabapentin is effective [46–49]. Supraspinal PGB actions involve activation of the descending inhibitory noradrenergic system in the locus coeruleus [47,48]. Spinal gabapentin produces analgesic effects through mechanisms that do not elicit or depend on this supraspinal-evoked noradrenaline release [47,49]. Given the reciprocal nature of neural connections between serotonin-rich nuclei of the RVM and noradrenergic nuclei of the dorsolateral pontine tegmentum [32,38], it is also possible that direct PGB actions in the A5–A7 noradrenergic nuclei produce indirect effects on RVM neurones or vice versa, so PGB dually alters both descending serotonergic and noradrenergic controls to modulate spinal nociceptive hyperexcitability in naïve and pathological states [6]. In addition, direct spinal actions could secondarily reduce brainstem nociceptive processing.

In conclusion, PGB reduces spinal and brainstem activity, and its analgesic actions are not dependent on pathology in visceral pain. A common effect of PGB in visceral and somatic pain states entails a reduction in central nociceptive hyperexcitability. This may involve interactions with the balance of descending modulation from the brainstem, where enhanced descending facilitatory controls (or a reduction in inhibitory influences) produced by acute visceral stimuli or somatic insult produce a permissive physiological state of central excitability for PGB to exert inhibitory actions. This could also underlie the analgesic efficacy of PGB in other pain states that develop in lack of any clear peripheral insult, such as irritable bowel syndrome and fibromyalgia [26,44], and effects on limbic function may relate to anxiolytic actions [31]. Here, central plastic changes in the spinal cord and higher centres that modulate descending controls and contribute to central excitability could both amplify nociceptive transmission and produce interacting comorbidities. The hypersensitivities that develop consequently in tandem can potentially be neutralized by central PGB inhibitions of neural activity.

## Conflict of interest statement

The authors declare that there are no conflicts of interest in publication of this manuscript.
